# Supportive care needs among patients with breast and cervical cancer at a tertiary cancer treatment center in ethiopia: a cross-sectional study

**DOI:** 10.1038/s41598-026-67983-z

**Published:** 2026-09-02

**Authors:** Bedilu Deribe Derese, Eva Johanna Kantelhardt, Thomas Frese, Betty Ferrell, Muluken Gizaw, Sefonias Getachew, Abigiya Wondimagegnehu, Adamu Addissie, Eric Sven Kroeber, Lesley Taylor, Susanne Unverzagt

**Affiliations:** 1 Institute of General Practice and Family Medicine, Center for Health Sciences, Martin Luther University Halle-Wittenberg, Halle (Saale), Germany; 2 Institute of Medical Epidemiology, Biometry and Informatics, Center for Health Sciences, Martin-Luther-University Halle-Wittenberg, Halle (Saale), Germany; 3 College of Medicine and Health Science School of Nursing, Hawassa University, Sidama, Ethiopia; 4 Comprehensive Cancer Center, Department of Nursing Education and Research, City of Hope National Medical Center, Duarte, CA USA; 5 Department of Epidemiology and Biostatics, School of Public Health, College of Health Sciences, Addis Ababa University, Addis Ababa, Ethiopia; 6 Department of Gynecology, Medical Faculty, Martin Luther University Halle-Wittenberg, Halle (Saale), Germany; 7 Comprehensive Cancer Center, Department of Surgery, Division of Breast Surgery, City of Hope National Medical Center, Duarte, CA USA; 8 Global and Planetary Health Working Group, Center for Health Sciences, Martin-Luther-University Halle-Wittenberg, Halle (Saale), Germany

**Keywords:** Neoplasms, Support care need, Breast cancer, Cervical cancer, Ethiopia, Cancer, Health care, Oncology

## Abstract

The rising incidence of cancer diagnoses alongside improving survival rates in low- and middle-income countries (LMICs) has substantially increased the demand for comprehensive supportive care. This study aimed to assess the supportive care needs of patients diagnosed with breast or cervical cancer at Hawassa University Comprehensive Specialized Hospital Cancer Treatment Center, Ethiopia. A hospital-based cross-sectional study was conducted among 349 patients with breast or cervical cancer between December 2023 and May 2024. Data were collected using three validated instruments: the Supportive Care Needs Survey Short Form (SCNS-SF34), the Functional Assessment of Chronic Illness Therapy – Spiritual Well-Being scale (FACIT-Sp-12), and the European Organization for Research and Treatment of Cancer Quality of Life Questionnaire (EORTC QLQ-C30). All instruments were scored according to their respective guidelines. Multivariable linear regression was performed to identify factors independently associated with supportive care needs. Patients were relatively young, with a mean age of 42.5 ± 12 years for breast cancer and 49.5 ± 12 years for cervical cancer. The majority reported cancer-related symptoms, and nearly all patients indicated unmet supportive care needs in at least one domain. Greater supportive care needs were significantly associated with the use of traditional or spiritual healing practices (mean difference [MD] 9.15, 95% CI: 3.51–14.59), lower functional performance status (MD 5.53, 95% CI: 3.11–7.96), higher number of children (MD 1.95, 95% CI: 0.90–3.01), and higher educational attainment (MD 2.58, 95% CI: 1.22–3.94). Conversely, having health insurance was associated with significantly lower supportive care needs (MD − 3.78, 95% CI: −7.36 to − 0.19). Unmet supportive care needs were prevalent across all assessed domains: physical and daily living, psychological, sexual health, patient care, and health information underscoring a substantial gap in comprehensive cancer care in this setting. To address these needs, healthcare systems should prioritize patient-provider communication, strengthen social support networks for high-need patients, integrate spiritual well-being into care planning, and develop context-sensitive, multidisciplinary supportive care strategies.

**Supplementary Information:**

The online version contains supplementary material available at https://doi.org/10.1038/s41598-026-67983-z.

## Introduction

Reductions in the death rate from non-communicable diseases, such as cancer, are crucial markers for achieving the sustainable development goal (SDG)^1,2^. Globally, cancer is among the leading causes of death from non-communicable diseases. In 2022, breast and cervical cancers accounted for over one million deaths worldwide^3^.

Supportive care encompasses five interconnected areas of care: information, psychological, spiritual, social, and physical. Unmet supportive care needs and challenges linked to cancer or its treatment can lead to unnecessary suffering for patients and their families, potentially compromising the standard of care and disease progression. Social isolation, stress, untreated mental health issues, and other factors may trigger emotional distress, hinder social responsibilities, impede treatment compliance, and may be associated with adverse health outcomes such as change in physiology and course of a disease process^4–6^.

Despite global advancements in breast and cervical cancer survival, sharp disparities in morbidity and treatment access persist in low-income countries due to systemic barriers like limited diagnostic facilities, high costs, and delayed presentation. Consequently, these inequities compound the severe psychosocial and existential disruptions faced by patients, who frequently struggle with anxiety, depression, and a loss of daily functioning^7–9^. Building on the healthcare disparities in low-income settings, a cancer diagnosis also profoundly disrupts patients’ psychosocial and spiritual functioning, frequently inducing anxiety, insomnia, depression, and existential struggles. As a growing survivor population increases the demand for supportive care, addressing these needs becomes critical; helping patients find meaning and regain control significantly reduces psychological distress and enhances their overall quality of life^10–12^.

A cancer diagnosis frequently triggers profound existential and spiritual struggles, yet patients who find meaning in their experience demonstrate enhanced mental health, greater adaptability, and reduced psychological distress^9,13–15^. Given the growing population of cancer survivors, addressing these spiritual dimensions through structured supportive care is vital to mitigating the psychological burden and improving overall quality of life^16,17^.

While spiritual beliefs profoundly influence treatment-seeking behaviour, medical decision-making, and coping mechanisms either offering psychological relief or causing distress through views like divine punishment spiritual care in oncology settings is often treated as optional. Given the scarcity of African-context research on these psychosocial and spiritual dimensions^18^.

In Ethiopia, women with breast and cervical cancer experience multifaceted supportive care needs driven by societal stigma, financial strain, and severe psychosocial burdens such as fear of recurrence and treatment side effects all compounded by daily household responsibilities^19^. This burden is worsened by complex diagnostic journeys and low health literacy, which induce high stress and prompt patients to delay medical care in favour of traditional or religious healing^20^.

Prior local research on supportive care needs faced methodological constraints potentially limiting the generalizability of the study, particularly regarding adherence to standardized scoring protocols and the adaptational validation of assessment tools within the local context. This study addresses these limitations by focusing on a targeted population of breast and cervical cancer patients in Southern Ethiopia. By utilizing the Short-Form Supportive Care Needs Survey (SCNS-SF34) in strict alignment with its established scoring guidelines, this research provides a contextually validated and methodologically sound assessment to accurately quantify unmet holistic needs and identify their associated factors.

## Method and materials

### Setting and study period

The study was conducted at Hawassa University Comprehensive Specialized Hospital Cancer Treatment Centre (HUCSH-CTC) in Hawassa city, one of six cancer treatment centres in the country. As the only academic hospital offering comprehensive cancer care in Southern Ethiopia, it serves people living in the southern Oromia, Sidama region, central Ethiopia, and southern Ethiopian regions, making the total population 20–25 million people with diverse socio-cultural and economic backgrounds. Common cancers treated at the center and in Ethiopia include breast, cervical, gynaecological and colorectal cancers, non-Hodgkin’s lymphoma, and leukaemia^21^. Breast and cervical cancers are the most common cancers treated at the centre. Like other Ethiopians, patients at this cancer centre rely on agriculture for their livelihoods and mostly pay for healthcare out of pocket. The center has started radiotherapy treatment since May 2024.

### Design and population

This cross-sectional study was conducted at HUCSH-CTC from December 25th 2023 to May 25th 2024 and included all female adult patients receiving treatment for breast and cervical cancer. The study excluded patients who had transferred in after undergoing more than one cycle of chemotherapy at other centres. Patients who had previously received care at other cancer or chemotherapy centres were excluded to avoid confounding effects on their perceptions of supportive care needs, quality of life, and spiritual well-being that were influenced by their original treatment center. Additionally, patients who were unable to communicate were excluded due to their inability to respond accurately to questions assessing their supportive care needs. Communication difficulties included those caused by physical or medical conditions.

To ensure maximum representativeness, generalizability, and statistical power, the sample size was determined by evaluating multiple localized study parameters, including individual supportive care domains and psychosocial distress. The baseline prevalence of psychosocial distress was ultimately selected as the anchoring parameter because it yielded the maximum, most conservative sample size, safeguarding the study against being underpowered for both descriptive and multivariable analyses. This approach was particularly necessary given the methodological heterogeneities and varying scoring frameworks observed in prior localized supportive care literature. Assuming a single population proportion formula, the sample size was calculated using an estimated prevalence of 64% for psychosocial distress among cancer patients in Ethiopia, derived from a previous study in Addis Ababa^22^ utilizing a 95% confidence interval and a 5% margin of error, the initial required sample size was determined to be 354. After factoring in a 5% non-response rate to account for potential missing data or attrition, the final total sample size was calculated to be 372 patients.

### Data collection procedure and quality assurance technique

A list of 760 patients with breast and cervical cancer receiving treatment at HUCSH-CTC was compiled and used as a sample frame. The medical record number, which was taken from the patient’s registration chart, was used to complete the listing. The patient’s medical record verified the diagnosis. The Random Number Generator mobile app was then used to randomly select the patients.

A Mobile APP named Kobo-Collect was utilized for data collection. After verifying its functionality, it was deployed for a survey. Four nurses from HUCSH-CTC with experience in data collection were trained in a comprehensive 3-day training that covered the study’s objectives, questionnaire content, data collection methods, and ethical considerations. Data were gathered through face-to-face interviews with patients, who were selected, upon their arrival at the cancer treatment centre. Throughout data collection, the investigator (BD) closely supervised and monitored the process to ensure data quality. Any issues with data completeness or encountered difficulties were promptly addressed, leading to two updates of the mobile application tool version.

### Outcome measures and tools

The primary outcome was supportive care need. The secondary outcomes were spiritual wellbeing and quality of life.

Sociodemographic variables were collected through a structured interview questionnaire. Urban residency is applicable to patients from regions with a minimum population of 2,000 and the administrative body of town municipality^23^. Medical information on tumour entity, date of initial diagnosis and time since initial diagnosis, disease stage, information on remission, recurrence and progression of cancer, metastases, and current cancer treatments, and results of laboratory investigations were collected by structured medical record review format.

#### Supportive care needs assessment

To evaluate the supportive care needs of cancer patients, the Supportive Care Needs Survey Short Form (SCNS-SF34) was employed. This validated tool consists of 34 items that measure five distinct domains: psychological needs, health system and information needs, physical and daily living needs, patient care and support needs, and sexuality needs. The total score ranges from 34 to 170, with higher scores indicating greater levels of unmet supportive care needs. The SCNS-SF34 has been previously validated for use in Ethiopia, ensuring its cultural relevance and reliability (*r* = 0.93) in this context^24^. The total supportive care need score was treated as a continuous primary dependent variable for the regression analyses, per the instrument’s scoring guidelines. Prior to modelling, the assumption of normality was formally checked. Distribution symmetry was evaluated using skewness and kurtosis indices, with values within an acceptable threshold of *±* 2.0 considered indicative of a sufficiently normal distribution for parametric modelling.

#### Spiritual well-being measurement

Spiritual well-being was assessed using the Functional Assessment of Chronic Illness Therapy – Spiritual Well-Being Scale (FACIT-Sp-12). This instrument includes 12 items divided into three sub-domains: peace, meaning, and faith. These components allow for a comprehensive exploration of the spiritual dimensions of patient experience. The analysis incorporated both the two-factor model (peace/meaning and faith) and the three-factor model (peace, meaning, and faith). Scores range from 0 to 48, with higher scores reflecting better spiritual well-being which was translated to 15 different language and cultures found to be valid and reliable (α = 0.81–0.88)^25^.

#### Quality of life evaluation

Quality of life was measured using the European Organisation for Research and Treatment of Cancer Quality of Life Questionnaire (EORTC QLQ-C30). This widely recognized tool assesses multiple aspects of health-related quality of life in cancer patients, including physical, emotional, cognitive, and social functioning, as well as symptom burden and overall health status. It was valid and reliable to assess quality of life in patients with breast cancer (α = 0.7–0.81) and it is a robust and internationally validated instrument frequently used in oncology research^26,27^, .

### Data management and analysis

For the purposes of descriptive and inferential statistics, all of the gathered data was downloaded and exported to SPSS version 27.0 for Windows. The suggested score guidelines for each tool were implemented for domain analysis in quality of life, spiritual wellbeing, and supportive care needs. Descriptive statistics which included absolute and relative frequencies or means with standard deviations (SD) were utilized to summarize and contrast the quality-of-life and domain scores between breast and cervical cancer patient cohorts. Due to the marked imbalance in sample sizes between these two cancers, formal comparative inferential testing was intentionally avoided to mitigate the potential of violating comparative analytical assumptions. Therefore, descriptive approach was prioritized to accurately reflect the distinct clinical characteristics of each cohort without introducing over-interpreted or underpowered comparative metrics.

The primary dependent variable, total supportive care need score, was visually inspected for normal distribution using a histogram with an overlaid normal curve. Skewness and kurtosis assessments confirmed that the assumption of normality was satisfied; linearity, homoscedasticity, and multicollinearity diagnostics were similarly verified to ensure a robust model fit. Normality of the data was verified via visual inspection of normal probability (P-P) plots; the observed cumulative probabilities closely tracked the diagonal reference line, confirming that the regression standardized residuals conformed acceptably to a normal distribution. Homoscedasticity was confirmed through the visual appraisal of residual-versus-predicted value for scatterplots, the residual plot displayed a random rectangular pattern, indicating homoscedasticity. To assess for multicollinearity among the independent variables, the Variance Inflation Factor and tolerance values were computed. All variables retained in the final multivariable models demonstrated VIF < 5 confirming the absence of significant collinearity. Candidates for the multivariate regression model were variables from the simple linear regression model with a p value less than 0.25. We analysed the impact of various factors, such as sociodemographic and clinical characteristics, quality of life, and spiritual wellbeing, by employing a multivariate linear regression model while accounting for confounders. The overall explanatory power and fitness of the finalized model were robust, yielding a coefficient of determination (R2 = 0.413), indicating that the included independent variables accounted for 41.3% of the variance in the total supportive care need scores.

## Results

Data were collected from 362 patients with breast and cervical cancer during in-hospital treatment, but after data cleaning, 349 patients with complete information on the primary and secondary outcomes were included, giving a response rate of 93.8%. Of these, 305 were diagnosed with breast cancer and 44 with cervical cancer.

### Sociodemographic characteristics

The mean age was nearly 42.5 *±* 12 years for patients with breast cancer while it was 49.5 *±* 12 years for patients with cervical cancer. The youngest patient was 20 years old, while the oldest was 90 years old and a substantial percentage of patients younger than 40-years. In terms of the residence, almost 63% of patients lived in urban areas.

Almost 70% of people were from families with five or more members. The majority identified as Protestants (47.9%), with Orthodox (31.2%) and Muslims (20.9%). Most patients (93.1%) were married. Their level of education ranged from no formal education (17.5%) to college and above (24.1%). A reported 3874 Birr (SD 3138) was the mean monthly income which is within the lowest salary for public servants, and a majority (60.5%) earn less than $2.15 a day. More than 72% of patients have community-based health insurance (CBHI) (Table 1).

**Table 1:** Sociodemographic characteristics of patients with breast and cervical cancer attending HUCSH-CTC.

Characteristics		Breast cancer (*n* = 305)	Cervical cancer (*n* = 44)
Age in years (mean *±* SD)		42.5 *±* 12.1	49.5 *±* 12.3
Age range (years)		20 to 90	27 to 85
Age Group (years)	*≤* 39	135(44.3)	10(22.7)
	40–64	153(50.2)	27(61.4)
	≥ 65	17(5.6)	7(15.9)
Residence	Urban	191(62.6)	28(63.6)
	Rural	114(37.4)	16(36.4)
Family size	Up to four	100 (32.8)	12(27.3)
	Five or more	205(67.2)	32(72.7)
Religion	Islam	69(22.6)	4(9.1)
	Orthodox	96(31.5)	13(29.5)
	Protestant	140(45.9)	27(61.4)
Marital status	Single/divorce/ widowed	23(7.6)	1(2.3)
	Married	282(92.5)	43(97.7)
Educational status	No formal education	47(15.4)	14(31.8)
	Read and write	50(16.4)	14(31.8)
	Primary education	46(15.1)	7(15.9)
	Secondary education	82(26.9)	5(11.4)
	College and above	80(26.2)	4(9)
Occupation	Farmers/daily labour and others	37(12.1)	2(4.5)
	Government or professional	76(24.9)	7(15.9)
	Merchant	66(21.6)	6(13.6)
	Housewife	126(41.3)	29(65.9)
Income	Income (birr) (mean ± SD)	4041 *±* 3267	2716 *±* 1640
	Below $2.15 per day	175(57.4)	36(81.8)
CBHI		220(72.1)	32(72.7)
Living with	Family (husband, children)	227(90.8)	38(86.4)
	Other relative	28(9.2)	6(13.6)

CBHI: Community based health insurance; SD: standard deviation.

$2.15 per day is equivalent to 3677 Ethiopian birr in month.

### Clinical characteristics and symptoms

Patients reported various degrees of symptoms during the last 7 days, with some variation in symptoms in the two groups. The experience of symptoms associated with cancer varies, but approximately 86% of breast cancer patients and over 90% of cervical cancer patients reported suffering from lack of energy. In terms of pain, during the seven days before the interview, 89.5% of patients with breast cancer and nearly 93% of patients with cervical cancer reported experiencing pain of various self-reported severity. Patients’ treatment options were mainly chemotherapy and combinations of chemotherapy and surgery in patients with breast and cervical cancer (64 vs. 48%). More than 13% of patients sought spiritual and traditional treatments. In terms of patients’ functional status, a considerable majority had an ECOG Grade of 0 or 1 (Table 2).

**Table 2:** Clinical and reproductive health associated characteristics of breast and cervical cancer patients at HUCSH-CTC.

Characteristics		Breast cancer (*n* = 305)	Cervical cancer (*n* = 44)
Symptoms*: Lack of energy	Not at all	43(14.1)	4(9.1)
	A little bit	100(32.8)	9(20.5)
	Some what	116(38.0)	15(35.1)
	Very much	46(15.1)	16 (36.3)
Symptoms*: Pain	Not at all	32(10.5)	3(6.8)
	A little bit	129(42.3)	12(27.3)
	Some what	99(32.5)	17(38.6)
	Very much	45(14.6)	12(27.3)
Symptoms*: Treatment Side effects	Not at all	34(11.1%)	2(4.5)
	A little bit	64(21.0)	6(13.6)
	Some what	108(35.4)	17(38.6)
	Very much	99(32.5)	19(43.1)
Symptoms*: Time spent in bed	Not at all	139(45.6)	20(45.5)
	A little bit	101(33.1)	9(20.5)
	Some what	50(16.4)	13(29.5)
	Very much	15(5.0)	2(4.5)
Symptoms*: Satisfied with sexual life	Not at all	62(20.3)	13(29.5)
	A little bit	82(26.9)	7(15.9)
	Some what	59(19.3)	4(9.1)
	Quite	101(33.5)	20(45.0)
Stage of cancer	Stage 1	13(4.3)	0
	Stage 2	67(22.3%)	6(13.6%)
	Stage 3	178(59.1%)	27(61.4%)
	Stage 4	43(14.3%)	11(25.0%)
Duration of illness in years (mean *±* SD)		2.19 *±* 1.45	2.23 *±* 1.79
Treatment used	Chemotherapy	65(21.3)	20 (45.5)
	Chemotherapy and surgery	197(64.6)	21(47.7)
	Spiritual and traditional care	43(14.1)	3(6.8)
ECOG Grade	ECOG Grade 0	147(48.2)	12(27.3)
	ECOG Grade 1	109(35.7%)	25(56.8%)
	ECOG Grade ≥ 2	49(16.1)	7(15.9)

*Measured over the last seven days.

### Quality of life

Quality of life was generally higher for patients with breast cancer than for cervical cancer patients with differences of more than 10 points in role functioning, cognitive and social functioning and global quality of life score. The highest quality of life was scored in the cognitive domain for both patients with breast and cervical cancer (85 vs. 75 of 100 points). The lowest score (44 vs. 34 of 100 points) was observed in the social functioning domain (see Table 3).

**Table 3:** Domains of quality of life among patients with breast and cervical cancer attending HUCSH-CTC.

QOL Domains (0 to 100 points)	Breast cancer (*n* = 305)	cervical cancer (*n* = 44)
Physical Functioning	71(19.6)	69.24(17.3)
Role Functioning	70.4(26.2)	59.5(28.4)
Emotional Functioning	64.5(21.1)	59.7(18.8)
Cognitive Functioning	85.2(20.8)	74.6(31.2)
Social Functioning	44.3(23.7)	34.5 (22.9)
Symptom Scale	70.1(14.1)	65.22(17)
Global Health/QOL	65.3 (20)	55.7 (23)
Summary Score	72.5(14.6)	66.3(17)

### Spiritual wellbeing

**Fig. 1:**
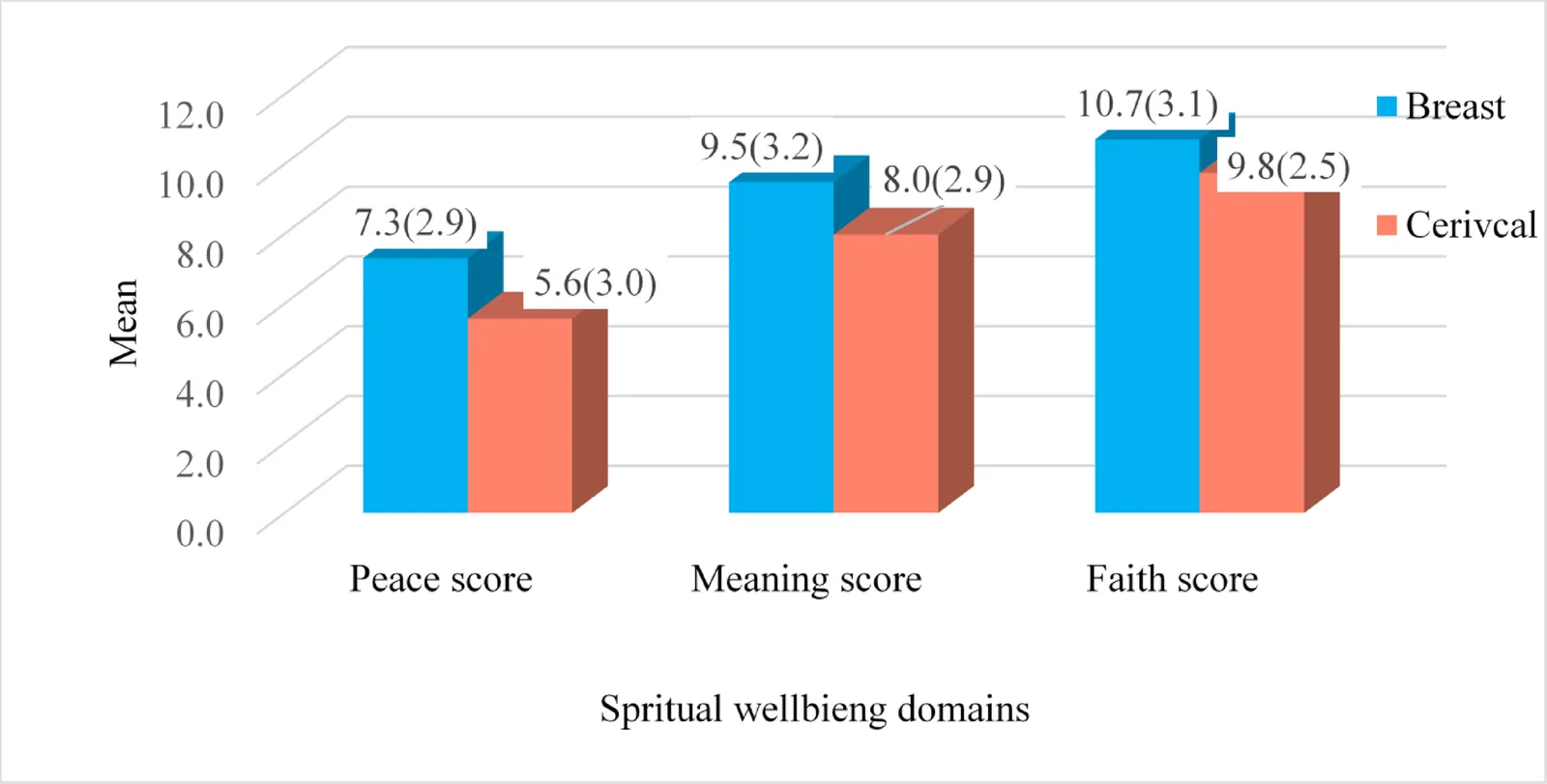
Domain based spiritual wellbeing mean score among patients with breast and cervical cancer attending HUCSH-CTC (*n* = 349).

Both cohorts scored relatively high in the faith domain of spiritual well-being, with patients with breast and cervical cancer scoring 10.7 *±* 3.1 or 9.8 *±* 2.5 of 16 points, respectively. In contrast, both cohorts had low peace scores: 7.3 for patients with breast cancer and 5.5 for those with cervical cancer. In the two-component analysis, the mean score for peace/meaning was 16.8 *±* 5.9 for breast cancer patients and 16.4 *±* 6.0 for cervical cancer patients. (See Fig. 1)

### Supportive care needs

**Fig. 2:**
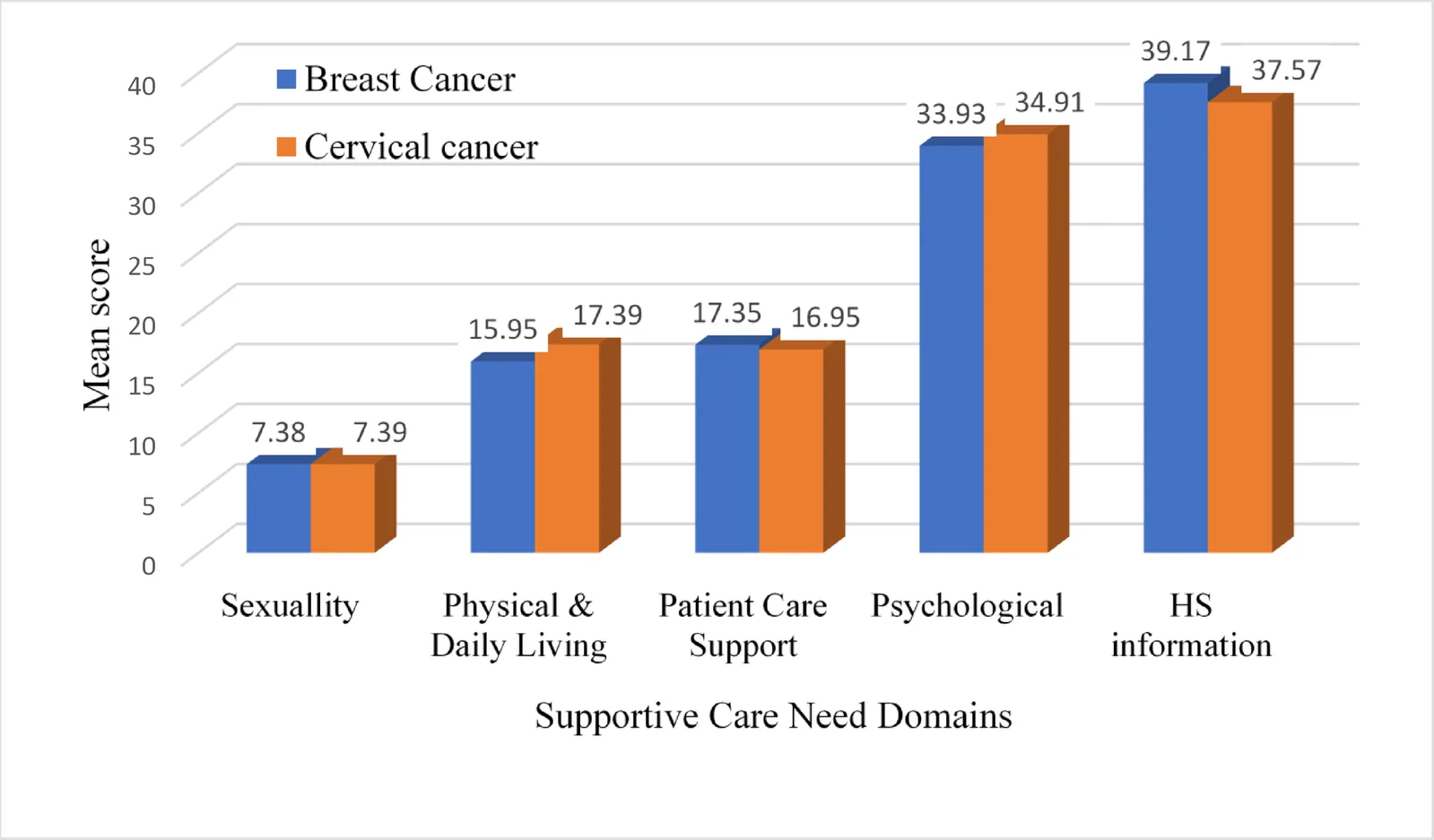
Patients with breast and cervical cancer attending HUCSH-CTC reporting at least some supportive care need (*n* = 349).

Over 90% of participants reported at least one unmet need in the supportive care domain. Nearly 98% of individuals with cervical and breast cancer reported unmet need in the domain of informational and health system. The specifics in this respect are given in Table 4; Fig. 2.

**Table 4:** Domain based supportive care need category among patients with breast and cervical cancer at Hawassa University Comprehensive Hospital Cancer Treatment Centre.

Supportive care need domains		Breast cancer (*n* = 305)	Cervical cancer (*n* = 44)
Patient Care and Support Need (5 questions)	No need	20 (6.6)	8 (18.2%)
	Some need	285 (93.4)	36 (81.8)
Sexuality Need (3 questions)	No need	27 (8.9)	1 (2.3)
	Some need	278 (91.1)	43 (97.7)
Psychological Need (10 questions)	No need	8 (2.6)	2 (4.5)
	Some need	297 (97.4)	42 (95.5)
Physical & Daily Living Need (5 questions)	No need	22 (7.2)	2 (4.5)
	Some need	283 (92.8)	42 (95.5)
Health system and information need (11 questions)	No need	6 (2)	2 (4.5)
	Some need	299 (98)	42 (95.5)

For patients with breast and cervical cancer, Fig. 2 details the supportive care score for each five domains of supportive care need. The mean (SD) total supportive care need score for the study population was 113.83 ± 17.83. Formal shape diagnostics yielded a skewness of -0.984 (SE = 0.131) and a kurtosis of 1.866 (SE = 0.260).

### Factors associated with supportive care need

**Fig. 3:**
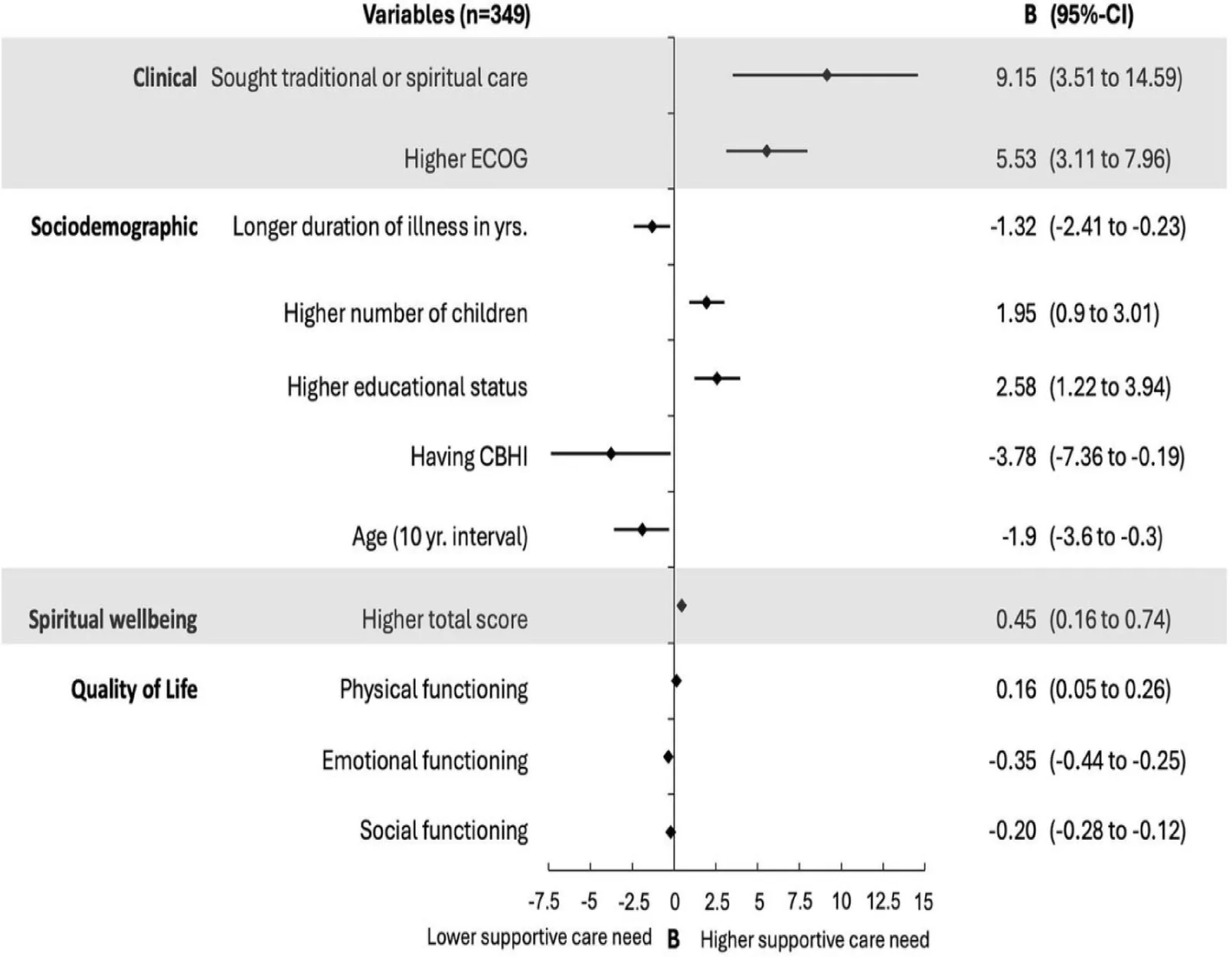
Factors associated with supportive care needs among patients with breast and cervical cancer attending HUCSH-CTC.

Total supportive care needs were higher for breast and cervical cancer patients who have already sought support from traditional or spiritual sources (MD 9.15; 95% CI: 3.51–14.59), patients with low performance status (high ECOG) (MD 5.53; 95% CI 3.11–7.96), higher number of children (MD 1.95; 95% CI 0.9–3.01) and higher educational status (MD 2.58; 95% CI 1.22–3.94). Patients with a community-based health insurance reported lower supportive care needs (MD -3.78, 95% CI -7.36 – -0.19). Patients who received spiritual and conventional care had a nearly nine-point greater demand for supportive care than those who had only chemotherapy (Fig. 3).

## Discussion

This study assessed the supportive care needs among breast and cervical cancer patients at a tertiary cancer treatment center in Southern Ethiopia. As stated in previous literature, cancer related symptoms, QoL, and spiritual wellbeing, community-based health insurance, and number of children were linked to supportive care needs. This study provided timely insights which can be used to guide the development of supportive care programs at HUSCH-CTC for patients throughout the continuum of their disease.

Our study found that patients diagnosed with breast and cervical cancer experience a wide range of symptoms at varying intensities, with over 90% reporting fatigue, pain, and side effects from treatment. Additionally, nearly 55% experienced periods of immobility, requiring bed rest for part of the week. These findings highlight the significant physical and emotional challenges faced by these patients and emphasize the need for tailored symptom management and comprehensive supportive care, aligning with results from cancer related fatigue studies^28^ and other studies on cancer related impairments and functional limitations^29^.

In our study patients exhibited higher faith scores in their spiritual well-being a key source coping compared the peace and meaning domains. Specifically, they scored 10.5 out of 16 points in the faith domain, but only 16.4 out of 32 in the combined peace-meaning domain, indicating a need for interventions that enhance overall spiritual well-being, particularly in the peace-meaning area. This result is consistent with a study from the United States^30^, but differs from a study in Japan^31^. The high faith scores may reflect the 99.9% religious affiliation in Ethiopia, as faith in God is a significant aspect of religiosity. The lower scores in the peace and meaning domains might stem from challenges like fatigue, pain, and unmet needs. Additionally, while meaning and peace correlate negatively with emotional well-being, they positively impact physical health being a means of cooping, influencing the supportive care needs of cancer patients^32–34^.

According to our study, patients with breast or cervical cancer have unmet needs for supportive care in a number of different areas. More than 90% of these patients said that they required supportive care on varying levels, with health information being the most in-demand. This finding is consistent with several studies showing that the majority of cancer patients require supportive care^35–38^. The high requirements for health system and information may indicate the mismatch between available services and patients’ informational needs related to cancer^39^. Providing patients with essential information can help alleviate their stress and reduce supportive care need in the domain of health system and information. Flexible access to clear, high-quality information resources is crucial in addressing patients’ needs and positively influencing their discomfort, anxiety, and depression related to supportive care through digital resources and assistance^37^.

In our study, cancer patients who utilized traditional and spiritual interventions exhibited higher supportive care needs. Contextually, patients frequently sought these options sequentially rather than concurrently with medical care, pausing or discontinuing conventional treatment before, during, or after its initiation. This lack of parallel care results in treatment discontinuity and missed medical appointments, aligning with their elevated supportive needs. Furthermore, our findings show that religiosity is closely linked to supportive care needs, consistent with literature highlighting the relationship between religious practice and patient requirements. This underscores that integrating spiritual services within hospital care may enhance patient well-being, addressing emotional needs by fostering a supportive healthcare environment. Research in Africa has shown that relying solely on God for symptom disclosure can delay medical care^40^. A cancer diagnosis often brings emotional distress and uncertainty about prognosis, negatively affecting the peace and meaning aspects of spiritual well-being and resulting in unmet supportive care needs, emphasizing the importance of integrating religious support with medical care^41–43^.

One out of every eight patients in our study had an impaired performance status with an ECOG of two or above and higher supportive care demands. Poor performance status among cancer patients is not only a key determinant of prognosis but also influences the decision-making process regarding supportive care^44^. Patients with better health status (lower ECOG) often experience milder symptoms, value independence, prefer cancer-suppressing treatments over symptom management, and may have higher hope of cure without supportive care. This finding was supported by a study conducted in Germany and Asian counties confirmed poor functional status as a key factor for unmet supportive needs among patients^45^.

Higher educational status in women with breast and cervical cancer was associated with increased supportive care needs, as evidenced by a study in Turkey^46,47^. Possible explanations for this relationship may be higher health awareness and health literacy among more educated women.

Supportive care requirements may vary based on the type of cancer^48^. Supportive care needs were higher in breast cancer patients compared to those with cervical cancer, possibly influenced by variations in treatment intensity, stage at the commencement of treatment and demographic factors such as their younger age and higher family size^49^. While our study found that patients with breast cancer demonstrated a comparatively higher need for supportive care than those with cervical cancer, this outcome reflects a direct comparison within the study population and should not be interpreted as minimizing the needs of cervical cancer patients. On the contrary, our findings also underscore a substantial demand for supportive care among individuals with cervical cancer. This is consistent with existing literature^50,51^, which emphasizes the considerable physical, emotional, and psychosocial burdens faced by these patients. Therefore, although breast cancer patients may show elevated needs in specific areas, the supportive care requirements of cervical cancer patients remain critically important. These findings—both from our research and from broader evidence—reinforce the necessity of prioritizing supportive care for cervical cancer patients, particularly during chemotherapy, to ensure comprehensive and equitable care.

In our study community-based health insurance was associated with lower supportive care need scores of patients. Health insurance is associated with transitioning from out-of-pocket payments to prepayment being crucial for alleviating financial burdens. It offers a promising alternative for enhancing access to primary healthcare by reducing financial barriers^52,53^. In our study, 75% of patients were enrolled in a CBHI system; however, it does not cover expensive drugs from private pharmacies, nor does it cover services outside their permanent residence. These findings highlight the necessity of revising health insurance schemes to address out-of-pocket healthcare expenses effectively.

### Strength and limitation

The thorough evaluation of supportive care needs along the course of cancer therapy provided important insights into how people manage the complex problems of cancer care by methodically assessing many sides of patient requirements. The quality of this study was greatly increased by using electronic data collection techniques. This method not only guarantees the accuracy and consistency of the data gathered, which are frequently present in manual collecting procedures, but it also makes it easier to integrate new technology. Effective data management and real-time updates are made possible by electronic systems, which is especially helpful in a subject that is changing quickly, like cancer therapy. The utilization of locally validated tools guaranteed that the data gathered appropriately reflects the supportive care needs of cancer patients.

On top of that, the multivariable linear regression model demonstrated an R^2^ value of 0.413, indicating that the included predictors accounted for 41.3% of the variance in total supportive care needs. In behavioural and psychosocial health research, outcome metrics are inherently driven by a complex web of individual behaviours, environmental variations, and unmeasured clinical factors. While physical sciences demand very high explanatory values, recent clinical literature confirms that an R^2^ exceeding 15% is considered a highly meaningful threshold for multifactorial health outcomes^54^. Therefore, our model’s explanatory power of 41.3% represents an exceptionally strong and robust model fit that captures a substantial portion of clinical variance while remaining safely free from the risks of artificial statistical overfitting.

However, a notable limitation of this study is the inherent difficulty in establishing cause-effect relationships due to its design to explain the association between supportive care needs and the use of traditional or spiritual care. Additionally, the relatively small number of participants in the cervical cancer group and their differences in stage of the disease and resulting treatment poses another challenge, as it may limit the statistical power to detect significant differences or trends within this subset.

Besides, this study combined breast and cervical cancer patients within the primary multivariable analyses despite the inherently distinct clinical, demographic, and psychosocial profiles characteristic of each malignancy. While our sample’s distribution directly mirrors the current country-level incidence in Ethiopia where cervical cancer accounts for approximately 15% of female oncological diagnoses the resulting asymmetry in subgroup sizes limited our ability to perform robustly powered, fully stratified comparative analyses without risking model fitting. Consequently, nuanced variations in supportive care needs specific to cervical cancer patients may be obscured. Future multi-site investigations should consider targeted oversampling strategies for specific gynaecological cohorts to facilitate independent, stratified predictive modelling.

## Conclusion

Ethiopian patients with breast and cervical cancer have high needs for supportive care. Health workers can identify patients probably having a higher need for supportive care: patients with impaired health status (ECOG), those who consult traditional healers and patients having a higher number of children. Especially these patients’ groups should prompt action to inquire needs and involve e.g., social workers on a routine basis. Addressing the supportive care needs of all breast and cervical cancer patients requires a holistic, multifaceted approach. Prioritizing effective communication with healthcare providers, strengthening social support systems and recognizing the importance of spiritual well-being in cancer care. Furthermore, we recommend interventional studies to evaluate the effect of implementing integrative treatment strategies in supportive care need of patients with breast and cervical cancer. Furthermore, with a strong emphasis on addressing health information needs within healthcare systems, which may help clinicians to identify needs of patients with breast and cervical cancer. However, interventional studies are required to confirm effectiveness of addressing these informational and health system needs of patients with breast and cervical cancer.

## Supplementary Information

Below is the link to the electronic supplementary material.

## Data Availability

The datasets used and analyzed during the current study are available from the corresponding author on reasonable request.
