# Supplementary material for: Supportive care needs among patients with breast and cervical cancer at a tertiary cancer treatment center in ethiopia: a cross-sectional study
Source: Sci Rep. 2026 Sep 2;16:27555. doi: 10.1038/s41598-026-67983-z (PMC13538695; doi:10.1038/s41598-026-67983-z)
Supplement: Supplementary file 1 [file 41598_2026_67983_MOESM2_ESM.pdf]

PROVIDING A VOICE FOR PATIENTS WORLDWIDE

FUNCTIONAL ASSESSMENT OF CHRONIC ILLNESS THERAPY (FACIT) LICENSING AGREEMENT

The Functional Assessment of Chronic Illness Therapy System of Quality of Life questionnaires and all related subscales, translations, and adaptations ("FACIT System") are owned and copyrighted by David Cella, Ph.D. The ownership and copyright of the FACIT System resides strictly with Dr. Cella. Dr. Cella has granted FACIT.org ("Licensor") the right to license usage of the FACIT System to other parties. Licensor represents and warrants that it has the right to grant the License contemplated by this agreement to the party listed below ("Licensee") for use of the measure and languages listed below in the study listed below ("Study"). This license is applicable for individual and/or academic researchers working on a not-for-profit research project.

Name ("Licensee"): Bedilu Deribe Derese

Measurement: FACT-Sp-12

Language(s): English

Study Title ("Study"): Supportive Care Needs Among Patients with Breast and Cervical Cancer at a Tertiary Cancer Treatment Center in Ethiopia: A Cross-Sectional Study

This current license is only extended to Licensee's Study subject to the following terms:

1) Licensee agrees to provide Licensor with copies of any publications resulting from this study or produced as a result of collecting data with any FACIT questionnaire.

2) Due to the ongoing and evolving nature of questionnaire development, treatment modalities and cross-cultural linguistic research, Licensor reserves the right to make adaptations or revisions to wording in the FACIT, and/or related translations as necessary. If such changes occur, Licensee will have the option of using either previous or updated versions according to their own research objectives.

3) Licensee may not change the wording or phrasing of any FACIT document without previous permission from Licensor. If any changes are made to the wording or phrasing of any FACIT item without permission, the document cannot be considered the FACIT, and subsequent analyses and/or comparisons to other FACIT data will not be considered appropriate. Permission to use the name "FACIT" will not be granted for any unauthorized translations of the FACIT items. Any analyses or publications of unauthorized changes or translated versions may not use the FACIT name. Any unauthorized translation will be considered a violation of copyright protection.

4) In all publications and on every page of the FACIT used in data collection, Licensor requires the copyright information be listed precisely as it is listed on the questionnaire itself. Furthermore, Licensee grants Springer Nature Limited non-exclusive authorization to publish the material in print and digital provided Springer Nature Limited indicates that the FACIT-Sp-12 is copyrighted by David Cella, Ph.D. and permission to use the FACIT-Sp-12 must be obtained from www.facit.org. While the copyright holders of the FACIT-Sp-12 material will be fully credited in the manuscript, the final publication will be published under a Creative Commons Attribution 4.0 International License (CC BY 4.0).

5) This license is not extended to electronic data capture by third party vendors of Licensee. Electronic versions by third party vendors of the FACIT questionnaires are considered derivative works and are not covered under this license. Permission for a third party to migrate and administer the FACIT electronically must be covered under separate agreement between the electronic data capture vendor and FACIT.org

6) In no case may any FACIT questionnaire be placed on the internet without password protection. To do so is considered a violation of copyright.

7) Licensor reserves the right to withdraw this license if Licensee engages in scientific or copyright misuse of the FACIT system of questionnaires.

8) There are no fees associated with this license.

9) This license is effective for two years upon the date of signature. If Licensee requires an extension beyond this 2-year period, Licensee must contact Licensor and obtain an extension.

Bedilu Deribe Derese

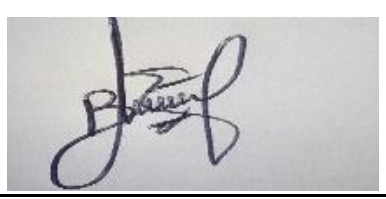

By:

Title: Assistant professor of adult health nursing, Phd Candidate

Date: 14/08/2026

FACIT.org

Digitally signed by Jason Bredle
Date: 2026.08.14 09:46:16
-05'00'

By: Jason Bredle

Title: Director, FACIT.org

Date: 14 August 2026
