# Supplementary material for: Supportive care needs among patients with breast and cervical cancer at a tertiary cancer treatment center in ethiopia: a cross-sectional study
Source: Sci Rep. 2026 Sep 2;16:27555. doi: 10.1038/s41598-026-67983-z (PMC13538695; doi:10.1038/s41598-026-67983-z)
Supplement: Supplementary file 2 [file 41598_2026_67983_MOESM1_ESM.doc]

**Data Collection Worksheet**

**Please Note:** The Data Collection Worksheet (DCW) is a tool to aid integration of a PhenX protocol into a study. The PhenX DCW is not designed to be a data collection instrument. Investigators will need to decide the best way to collect data for the PhenX protocol in their study. Variables captured in the DCW, along with variable names and unique PhenX variable identifiers, are included in the PhenX Data Dictionary (DD) files.

To help us plan better services for people diagnosed with cancer, we are interested in whether or not needs which you may have faced as a result of having cancer have been met. For every item on the following pages, indicate whether you have needed help with this issue within the last month as a result of having cancer. Put an x next to the number which best describes whether you have needed help with this in the last month. There are 5 possible answers to choose from:

| NO NEED | 1. Not applicable - This was not a problem for me as a result of not having cancer. |
| --- | --- |
|  | 2. Satisfied - I did need help with this, but my need for help was satisfied at the time |
| SOME NEED | 3. Low need - This item caused me concern or discomfort. I had little need for additional help |
|  | 4. Moderate need - This item caused me concern or discomfort. I had some need for additional help. |
|  | 5. High need - This item caused me concern or discomfort. I had a strong need for additional help. |

For example

In the last month, What was your level of need for help with:

2. Being informed about things you can do to help yourself to get well

| No need | | Some need | | |
| --- | --- | --- | --- | --- |
| Not applicable | Satisfied | Low need | Moderate need | High need |
| [ ] 1 | [ ] 2 | [ ] 3 | [X] 4 | [ ] 5 |

If you put the [x] where we have, it means you did not receive as much information as you wanted about things you could do to help yourself get well, and therefore needed some more information.

Now please complete the survey on the next 2 pages.

| In the last month, which was your level of need for help with: | No need | | Some need | | |
| --- | --- | --- | --- | --- | --- |
|  | Not applicable | Satisfied | Low need | Moderate need | High need |
| 1. Pain | [ ] 1 | [ ] 2 | [ ] 3 | [ ] 4 | [ ] 5 |
| 2. Lack of energy/ tiredness | [ ] 1 | [ ] 2 | [ ] 3 | [ ] 4 | [ ] 5 |
| 3. Feeling unwell a lot of the time | [ ] 1 | [ ] 2 | [ ] 3 | [ ] 4 | [ ] 5 |
| 4. Work around the home | [ ] 1 | [ ] 2 | [ ] 3 | [ ] 4 | [ ] 5 |
| 5. Not being able to do the things you used to do | [ ] 1 | [ ] 2 | [ ] 3 | [ ] 4 | [ ] 5 |
| 6. Anxiety | [ ] 1 | [ ] 2 | [ ] 3 | [ ] 4 | [ ] 5 |
| 7. Feeling down or depressed | [ ] 1 | [ ] 2 | [ ] 3 | [ ] 4 | [ ] 5 |
| 8. Feelings of sadness | [ ] 1 | [ ] 2 | [ ] 3 | [ ] 4 | [ ] 5 |
| 9. Fears about the cancer spreading | [ ] 1 | [ ] 2 | [ ] 3 | [ ] 4 | [ ] 5 |
| 10. Worry that the results of treatment are beyond your control | [ ] 1 | [ ] 2 | [ ] 3 | [ ] 4 | [ ] 5 |
| 11. Uncertainty about the future | [ ] 1 | [ ] 2 | [ ] 3 | [ ] 4 | [ ] 5 |
| 12. Learning to feel in control of your situation | [ ] 1 | [ ] 2 | [ ] 3 | [ ] 4 | [ ] 5 |
| 13. Keeping a positive attitude | [ ] 1 | [ ] 2 | [ ] 3 | [ ] 4 | [ ] 5 |
| 14. Feelings about death and dying | [ ] 1 | [ ] 2 | [ ] 3 | [ ] 4 | [ ] 5 |
| 15. Changes in sexual feelings | [ ] 1 | [ ] 2 | [ ] 3 | [ ] 4 | [ ] 5 |
| 16. Changes in your sexual relationships | [ ] 1 | [ ] 2 | [ ] 3 | [ ] 4 | [ ] 5 |
| 17. Concerns about the worries of those close to you | [ ] 1 | [ ] 2 | [ ] 3 | [ ] 4 | [ ] 5 |
| 18. More choice about which cancer specialists you see | [ ] 1 | [ ] 2 | [ ] 3 | [ ] 4 | [ ] 5 |
| 19. More choice about which hospital you attend | [ ] 1 | [ ] 2 | [ ] 3 | [ ] 4 | [ ] 5 |
| 20. Reassurance by medical staff that the way you feel is normal | [ ] 1 | [ ] 2 | [ ] 3 | [ ] 4 | [ ] 5 |
| 21. Hospital staff attending promptly to your physical needs | [ ] 1 | [ ] 2 | [ ] 3 | [ ] 4 | [ ] 5 |
| 22. Hospital staff acknowledging, and showing sensitivity to, your feelings and emotional needs | [ ] 1 | [ ] 2 | [ ] 3 | [ ] 4 | [ ] 5 |
| 23. Being given written information about the important aspects of care | [ ] 1 | [ ] 2 | [ ] 3 | [ ] 4 | [ ] 5 |
| 24. Being given information (written, diagrams, drawings) about aspects of managing your illness and side-effects at home | [ ] 1 | [ ] 2 | [ ] 3 | [ ] 4 | [ ] 5 |
| 25. Being given explanations of those tests for which you would like explanations | [ ] 1 | [ ] 2 | [ ] 3 | [ ] 4 | [ ] 5 |
| 26. Being adequately informed about the benefits and side-effects of treatments before you choose to have them | [ ] 1 | [ ] 2 | [ ] 3 | [ ] 4 | [ ] 5 |
| 27. Being informed about your test results as soon as feasible | [ ] 1 | [ ] 2 | [ ] 3 | [ ] 4 | [ ] 5 |
| 28. Being informed about cancer which is under control or diminishing (that is, remission) | [ ] 1 | [ ] 2 | [ ] 3 | [ ] 4 | [ ] 5 |
| 29. Being informed about things you can do to help yourself to get well | [ ] 1 | [ ] 2 | [ ] 3 | [ ] 4 | [ ] 5 |
| 30. Having access to professional counseling (eg, psychologist, social worker, counsellor, nurse specialist) if you, family or friends need it | [ ] 1 | [ ] 2 | [ ] 3 | [ ] 4 | [ ] 5 |
| 31. To be given information about sexual relationships | [ ] 1 | [ ] 2 | [ ] 3 | [ ] 4 | [ ] 5 |
| 32. Being treated like a person not just another case | [ ] 1 | [ ] 2 | [ ] 3 | [ ] 4 | [ ] 5 |
| 33. Being treated in a hospital or clinic that is as physically pleasant as possible | [ ] 1 | [ ] 2 | [ ] 3 | [ ] 4 | [ ] 5 |
| 34. Having one member of hospital staff with whom you can talk to about all aspects of your condition, treatment and follow-up | [ ] 1 | [ ] 2 | [ ] 3 | [ ] 4 | [ ] 5 |

Thank you for completing this survey

Protocol source: <https://www.phenxtoolkit.org/protocols/view/321101>
